# 2-Chlorohexadecanoic acid induces ER stress and mitochondrial dysfunction in brain microvascular endothelial cells

**DOI:** 10.1016/j.redox.2018.01.003

**Published:** 2018-01-05

**Authors:** Eva Bernhart, Nora Kogelnik, Jürgen Prasch, Benjamin Gottschalk, Madeleine Goeritzer, Maria Rosa Depaoli, Helga Reicher, Christoph Nusshold, Ioanna Plastira, Astrid Hammer, Günter Fauler, Roland Malli, Wolfgang F. Graier, Ernst Malle, Wolfgang Sattler

**Affiliations:** a Gottfried Schatz Research Center for Signaling, Metabolism and Aging, Molecular Biology and Biochemistry, Medical University of Graz, Austria; b BioTechMed Graz, Austria; c Institute of Physiological Chemistry, Medical University of Graz, Austria; d Gottfried Schatz Research Center for Signaling, Metabolism and Aging, Cell Biology, Histology and Embryology, Medical University of Graz, Austria; e Clinical Institute of Medical and Chemical Laboratory Diagnostics, Medical University of Graz, Austria

**Keywords:** HA, hexadecan-1-oic (palmitic) acid, HyA, hexadec-15-yn-1-oic acid, 2-ClHDA, 2-chlorohexadecan-1-al, 2-ClHDyA, 2-chlorohexadec-15-yn-1-al, 2-ClHA, 2-chlorohexadecan-1-oic acid, 2-ClHyA, 2-chlorohexadec-15-yn-1-oic acid, Apoptosis, Blood-brain barrier, Lipotoxicity, Myeloperoxidase, Neuroinflammation, Structured illumination microscopy

## Abstract

Peripheral leukocytes induce blood-brain barrier (BBB) dysfunction through the release of cytotoxic mediators. These include hypochlorous acid (HOCl) that is formed via the myeloperoxidase-H_2_O_2_-chloride system of activated phagocytes. HOCl targets the endogenous pool of ether phospholipids (plasmalogens) generating chlorinated inflammatory mediators like e.g. 2-chlorohexadecanal and its conversion product 2-chlorohexadecanoic acid (2-ClHA). In the cerebrovasculature these compounds inflict damage to brain microvascular endothelial cells (BMVEC) that form the morphological basis of the BBB. To follow subcellular trafficking of 2-ClHA we synthesized a ‘clickable’ alkyne derivative (2-ClHyA) that phenocopied the biological activity of the parent compound. Confocal and superresolution structured illumination microscopy revealed accumulation of 2-ClHyA in the endoplasmic reticulum (ER) and mitochondria of human BMVEC (hCMEC/D3 cell line). 2-ClHA and its alkyne analogue interfered with protein palmitoylation, induced ER-stress markers, reduced the ER ATP content, and activated transcription and secretion of interleukin (IL)−6 as well as IL-8. 2-ClHA disrupted the mitochondrial membrane potential and induced procaspase-3 and PARP cleavage. The protein kinase R-like ER kinase (PERK) inhibitor GSK2606414 suppressed 2-ClHA-mediated activating transcription factor 4 synthesis and IL-6/8 secretion, but showed no effect on endothelial barrier dysfunction and cleavage of procaspase-3. Our data indicate that 2-ClHA induces potent lipotoxic responses in brain endothelial cells and could have implications in inflammation-induced BBB dysfunction.

## Graphical abstract

**Figure:**
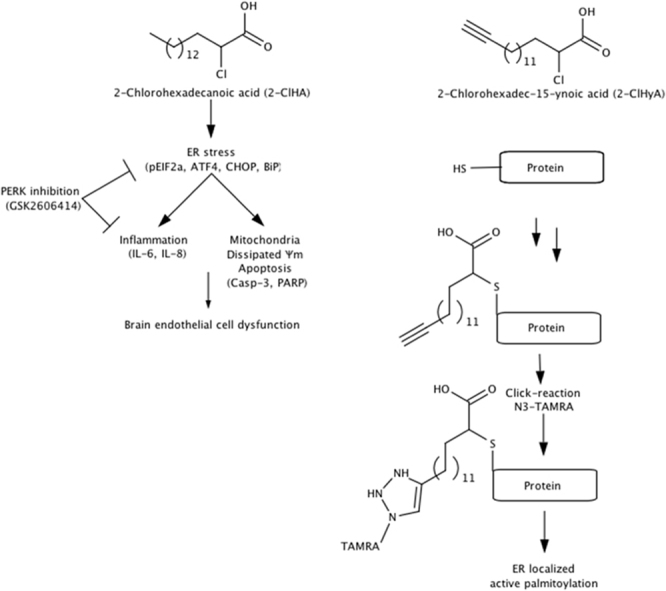

## 1 Introduction

The neurovascular unit separates most regions of the brain from the peripheral circulation to maintain the specialized central nervous system (CNS) micromilieu [1]. Brain microvascular endothelial cells (BMVEC) form the morphological basis of the blood-brain barrier (BBB) by the formation of tight junction (TJ) and adherens junction complexes [2]. These junctional complexes inhibit paracellular leakage and maintain CNS homeostasis via polarized expression of transporter systems taking a central biochemical gate-keeping function at the BBB [3], [4].

Under inflammatory conditions BBB function is compromised and can aggravate neuronal dysfunction [5]. Many of the pathways that interfere with BBB and neuronal function converge on the formation of reactive species [6]. This is of importance since TJ proteins are sensitive to alterations of the intracellular redox status [7] and oxidative stress induces a downregulation of the TJ protein occludin and disrupts the cadherin-catenin complex in brain endothelial cells [8]. In cerebrovascular diseases and stroke reactive oxygen species (ROS) can inhibit cerebral blood flow and impact barrier function [9], [10], [11], [12]. Additionally, oxidative stress-induced activation of matrix metalloproteinases (MMPs) and fluid channel aquaporins promote leakiness of the BBB and vascular edema [13].

During earlier work we could show pronounced BMVEC barrier dysfunction in response to the chlorinated fatty aldehyde 2-chlorohexadecanal (2-ClHDA) that is generated during endotoxemia [14], [15]. 2-ClHDA is formed through attack of plasmalogens (ether phospholipids) by hypochlorous acid/hypochlorite (HOCl/OCl^-^) [16], [17] generated via the myeloperoxidase (MPO)-H_2_O_2_-Cl^-^ system of activated phagocytes [18]. Under physiological conditions MPO is part of the innate immune system [19], however, under chronic inflammatory conditions MPO is considered a disease modifier [20]. MPO-derived oxidants have been shown to contribute to atherosclerosis and plaque instability [21], [22], [23], cardiac dysfunction [24], or diseases with a neuroinflammatory component [25]. The involvement of MPO in barrier dysfunction was also demonstrated during bacterial meningitis [26], [27]. MPO is expressed in demyelinated lesions in Multiple Sclerosis (MS) in humans and rodents [28]. In line, pharmacological inhibition of MPO reduced the severity of clinical symptoms in a murine MS model [29]. In response to systemic lipopolysaccharide (LPS) administration MPO levels in mouse brain are elevated and accompanied by decreased brain plasmalogen content and concomitant formation of 2-ClHDA [14]. In line with deleterious effects of MPO-generated 2-ClHDA [15], the MPO inhibitor *N*-acetyl lysyltyrosylcysteine amide ameliorates brain damage in a murine model of stroke [30] and counteracts BBB damage in a murine model of MS [31].

The electrophile 2-ClHDA impairs protein function by covalent modification, thereby triggering cytotoxic and adaptive responses that are typically associated with oxidative stress [32]. Consequently, conversion of (reactive) aldehydes to their corresponding alcohol and/or carboxylic acid analogues via the fatty alcohol cycle was considered as a protective pathway [33]. The Ford group has first demonstrated that 2-ClHDA is oxidized to 2-chlorohexadecanoic acid (2-ClHA) within this metabolic pathway [34]. The same group has shown that 2-ClHA accumulates in activated monocytes and induces apoptosis through ROS formation and endoplasmic reticulum (ER) stress pathways [35].

During the present study we synthesized and analytically characterized an alkyne derivative of 2-ClHA, namely 2-chlorohexadec-15-yn-1-oic acid (2-ClHyA) that is accessible to copper-catalyzed cycloaddition reactions. We investigated subcellular distribution using conventional confocal laser scanning microscopy and superresolution structured illumination microscopy (SIM), followed by characterization of ER- and mitochondria-specific cellular responses. Our data identify 2-ClHA as an inflammatory lipid mediator that interferes with protein palmitoylation and compromises ER- and mitochondrial functions in the human brain endothelial cell line hCMEC/D3.

## 2 Material and methods

A detailed Materials and Methods section describing synthetic and analytical procedures, cell culture, MTT and ECIS analyses, click-chemistry and subcellular colocalization studies [32], ATP measurements [36], metabolic labelling procedures, Western, FACS and qPCR analyses, ELISA and statistical data analysis is provided in the SUPPLEMENT.

## 3 Results

**Fig. 1:**
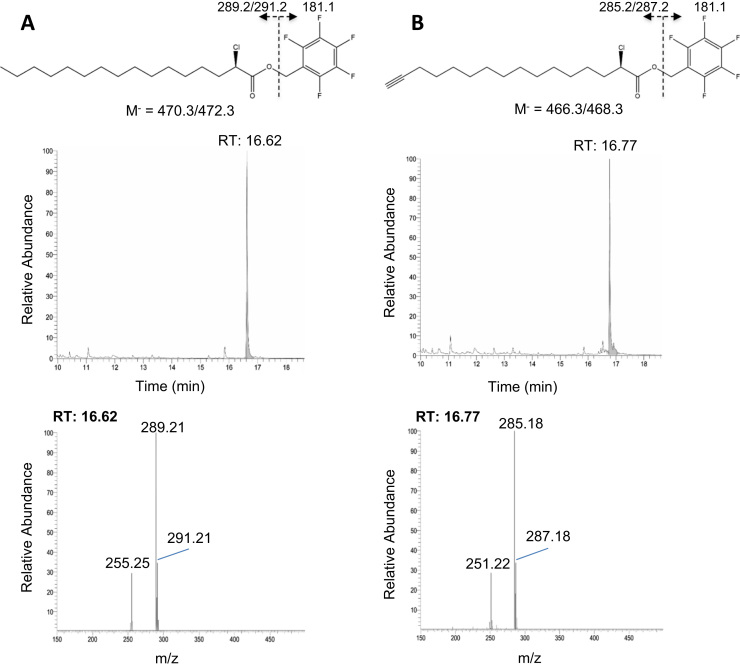
NICI-GC-MS characterization of the PFB-ester derivatives of 2-ClHA and 2-ClHyA. 2-ClHA and 2-ClHyA were converted to the corresponding PFB ester derivatives in acetonitrile containing *N,N′*-diisopropylethylamine. Structure and proposed fragmentation, elution profile, and NICI mass spectra of the (**A**) 2-ClHA and (**B**) 2-ClHyA PFB ester derivatives are shown in the upper, middle, and lower panels, respectively.

As a first step we synthesized 2-ClHA and 2-ClHyA containing a terminal alkyne group suitable as click-chemistry scaffold [32]. 2-ClHA and the clickable orthologue 2-ClHyA were synthesized from 2-ClHDA or 2-ClHDyA using oxone as the oxidant [37]. The resulting products were characterized by NICI-GC-MS as PFB-ester derivatives (structures given in Fig. 1**A** and **B**, upper panels). The PFB-esters eluted as single peaks under the analytical conditions used (Fig. 1A and B, middle panels). The molecular ions (M^-^) at m/z 469.9 and 465.9 were undetectable under the chromatographic conditions employed (Fig. 1**A** and **B**, lower panels). The intensity ratios of the diagnostic fragment ions at m/z 289.2/291.2 and 285.2/287.2 (proposed structures are shown in Fig. 1**A** and **B**, upper panels) of approx. 3:1 demonstrate the presence of two chlorine isotopes (^35^Cl/^37^Cl) in the analyte. The additional fragments at m/z 255.2 and 251.2 result from chlorine abstraction at C2 of the acyl fragments.

To ensure that 2-ClHA and its alkyne analogue 2-ClHyA display similar effects on hCMEC/D3 cell function MTT assays and impedance measurements were performed (to avoid trapping of chlorinated fatty acids by serum proteins all experiments were conducted with serum-starved cells). These experiments revealed significantly decreased MTT reduction at 8 and 24 h (Fig. S1A). Endothelial barrier function was monitored in real time using the ECIS system. While DMSO (vehicle) and HA (10 µM) was without effect, 2-ClHA and 2-ClHyA time dependently compromised barrier function (4 kHz) and monolayer integrity (64 kHz) in a comparable manner (Fig. S1B).

**Fig. 2:**
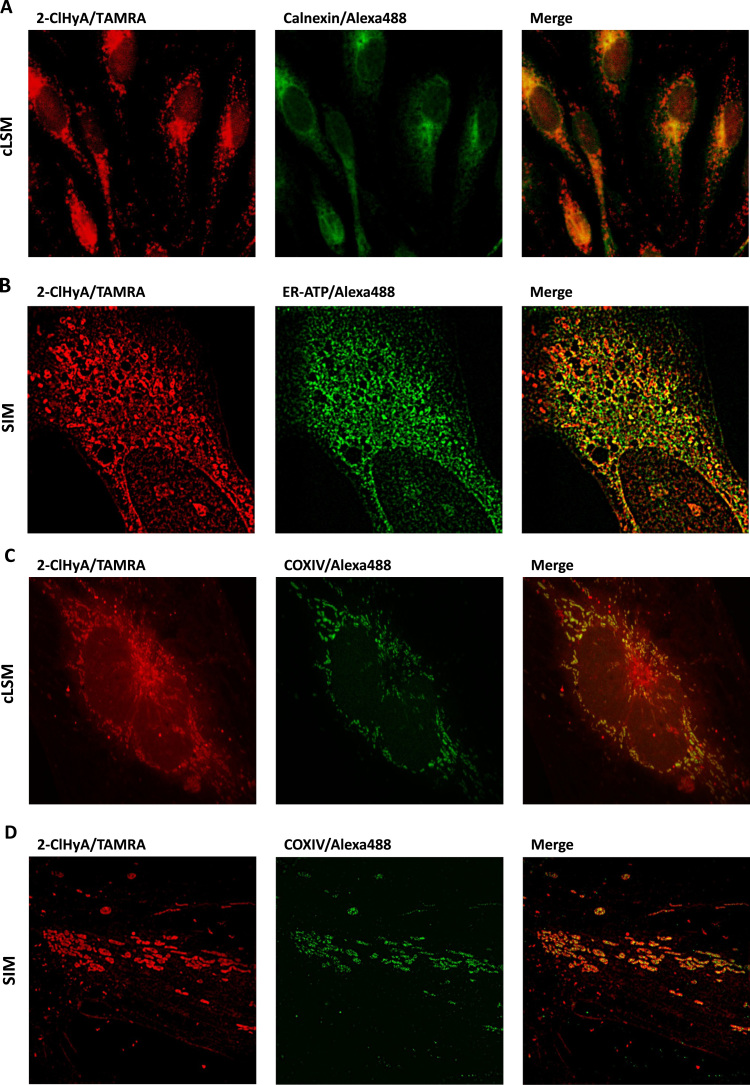
2-ClHyA is detected in the ER and mitochondria of hCMEC/D3 cells. **(A)** Cells were treated with 2-ClHyA (25 µM, 30 min), fixed with para-formaldehyde, permeabilized with Triton X-100 and clicked with N_3_-TAMRA. ER-labelling was performed with anti-calnexin antibody (1:100 in antibody diluent; 4 °C over night). Alexa 488-labelled goat anti-rabbit (1:300 in antibody diluent) was used as secondary antibody. Cells were visualized by cLSM. (**B**) Cells were transfected with a FRET-based ER-targeted ATP sensor plasmid (ERAT4.01) and then treated with 2-ClHyA as described in (A). Cells were fixed, permeabilized, clicked with N_3_-TAMRA and visualized by SIM. (**C, D**) Cells were treated with 2-ClHyA as described in (A) and then incubated with anti-COX IV antibody (1:100 in antibody diluent, 4 °C overnight). Alexa 488-labelled goat anti-rabbit (1:300 in antibody diluent) was used as secondary antibody. Cells were visualized by cLSM (C) or SIM (D).

Following copper-dependent cycloaddition with N_3_-TAMRA subcellular localization of 2-ClHyA was visualized by two approaches, namely cLSM and superresolution SIM. During cLSM mainly perinuclear colocalization of the 2-ClHyA-TAMRA adduct with Alexa488-labelled anti-calnexin (used as ER membrane marker) was observed (Fig. 2**A**). In more peripheral areas TAMRA fluorescence was also detected as isolated red fluorescence. Using superresolution SIM (partial) colocalization of the 2-ClHyA-TAMRA adduct with a genetically encoded ER-targeted fluorescent ATP sensor [36] (Fig. 2**B**). Also SIM data suggested that 2-ClHyA is not exclusively detected together with the ER marker. Indeed, cLSM revealed accumulation of 2-ClHyA in mitochondria as shown by colocalization of 2-ClHyA-TAMRA and Alexa-labelled anti COX IV (Fig. 2**C**). Superresolution SIM unveiled that 2-ClHyA-TAMRA accumulates at the inner mitochondrial membrane and within the matrix of mitochondria (Fig. 2**D**).

**Fig. 3:**
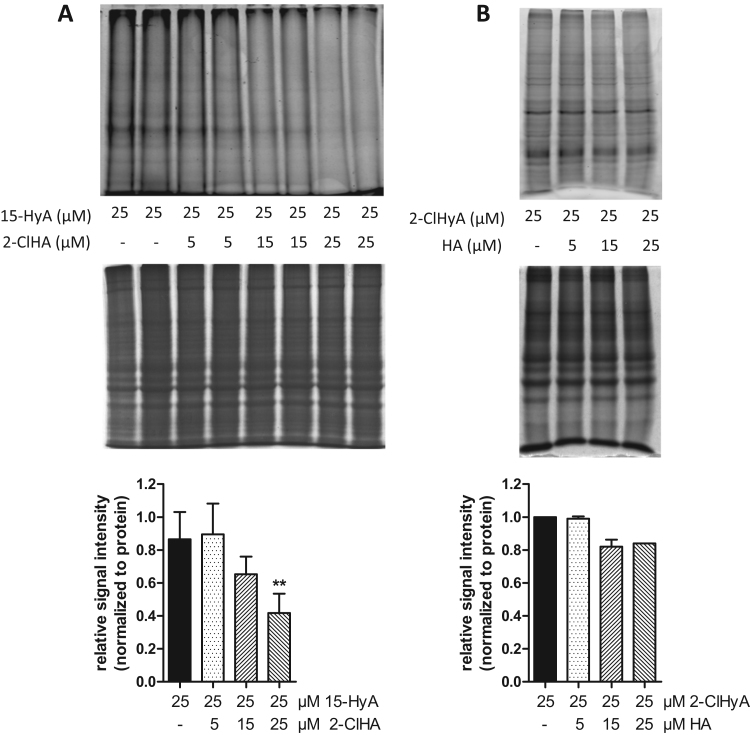
2-ClHA interferes with protein palmitoylation in hCMEC/D3 cells. Cells were metabolically labelled with (**A**) hexadec-15-yn-1-oic acid (HyA; 25 µM, 37 °C, 4 h; 2-ClHA was used as competitor) or (**B**) 2-ClHyA (25 µM, 37 °C, 4 h; HA was used as competitor). Cells were lysed and protein aliquots were subjected to click chemistry with N_3_-TAMRA, separated by SDS-PAGE, and visualized using a Typhoon 9400 scanner (upper panels). Coomassie Brilliant Blue staining was used to validate equal lane loading (middle panels). Metabolic labelling efficacy as a function of the corresponding competitor (fluorescence intensities are normalized to protein loading) is shown in the lower panels. Values are expressed as mean + SD. (**,p < 0.01; one-way ANOVA with Bonferroni correction).

We then moved on to establish consequences of 2-ClHA on ER- and mitochondrial function using hCMEC/D3 cells. Since 2-ClHA accumulates in the ER, we reasoned that 2-ClHA could affect protein-S-palmitoylation in a similar way as shown for 2-bromopalmitate (2-BrHA), a commonly used chemical inhibitor of protein palmitoylation [38]. To test this hypothesis cells were metabolically labelled with HyA (25 µM; Fig. 3**A**; upper panel). In the absence of 2-ClHA several protein bands of different molecular mass were labelled. Coomassie stained gels (to account for equal loading) are shown in the middle panels. Quantitation of fluorescence intensities (Fig. 3**A**; lower panel) indicates that in the presence of equimolar 2-ClHA concentrations protein acylation with HyA was reduced by 60% indicating that 2-ClHA interferes with protein palmitoylation. In a reverse experimental approach cells were labelled with 2-ClHyA (25 µM) and incorporation was competed with HA (5, 15, and 25 µM; Fig. 3**B**; upper panel). The Coomassie-stained gel is shown in the middle panel as loading control. In these experiments target protein labelling by 2-ClHyA was efficient but competition by HA was low (approx. 20% at 15 and 25 µM; Fig. 3**B** lower panel). This indicates covalent alkylation rather than formation of a labile thioester bond.

**Fig. 4:**
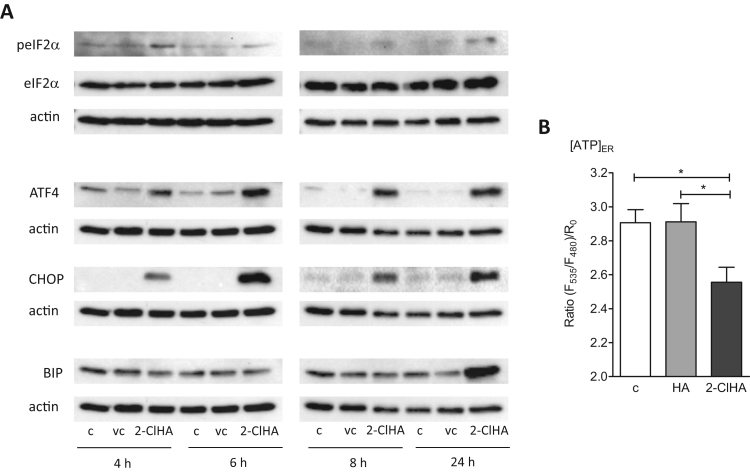
2-ClHA induces ER stress depletion of [ATP]_ER_ in hCMEC/D3 cells. (**A**) Serum starved cells were incubated in the absence (c) or presence of DMSO (0.1%, vehicle control, vc) and 2-ClHA (10 μM) for the indicated time points. Total cell lysates were subjected to SDS-PAGE. Antibodies against p-eIF2α, eIF2α, ATF4, CHOP, and BiP were used as primary antibodies. β-Actin was used as loading control. For each protein one representative blot out of three independent experiments is shown. Densitometric and statistical evaluation of Western blot results is shown in Fig. S2. (**B**) Cells expressing ERAT4.01 were cultured in the absence (c), or presence of HA (25 µM; 30 min) or 2-ClHA (both 25 µM, 30 min) and the FRET ratio was acquired on a Zeiss AxioVert inverted microscope. Columns represent ratio signals of ERAT4.01 obtained for the respective treatment conditions. At least 28 cells were analyzed in two independent experiments.

Aberrant protein palmitoylation induces ER stress, a pathway detrimental for brain endothelial barrier function [39]. Incubation of hCMEC/D3 cells with 2-ClHA resulted in phosphorylation of eIF2α starting 4 h post treatment, while total eIF2α levels remained unchanged (Fig. 4**A**). In line, expression of ATF4, a target gene of eIF2α, was increased by 2-ClHA when compared with untreated or vehicle-treated cells. In concert with eIF2α phosphorylation and upregulated ATF4 expression, 2-ClHA induced expression of the cell death executor CHOP. In parallel, expression of a central ER stress modulator and major chaperon, BiP (also termed GRP78) was upregulated after 24 h of cell treatment with 2-ClHA. Densitometric evaluation is shown in Fig. S2. Taken together, these results demonstrate that ER stress-related proteins are increased in response to 2-ClHA.

BiP contributes to protein folding by binding unfolded proteins in an ATP-dependent manner [40]. As 2-ClHA induced ER stress, we hypothesized that it also alters the ATP concentration within the ER lumen ([ATP]_ER_). To determine effects of 2-ClHA on [ATP]_ER_ in single cells we used a genetically encoded ER targeted, Förster resonance energy transfer (FRET)-based ATP probe (ERAT), which was developed recently [36]. These measurements revealed that 2-ClHA (but not HA) induced a decrease in the FRET ratio signal of ERAT by 40%, indicating significantly diminished [ATP]_ER_ in cells that were treated with 2-ClHA (Fig. 4**B**).

**Fig. 5:**
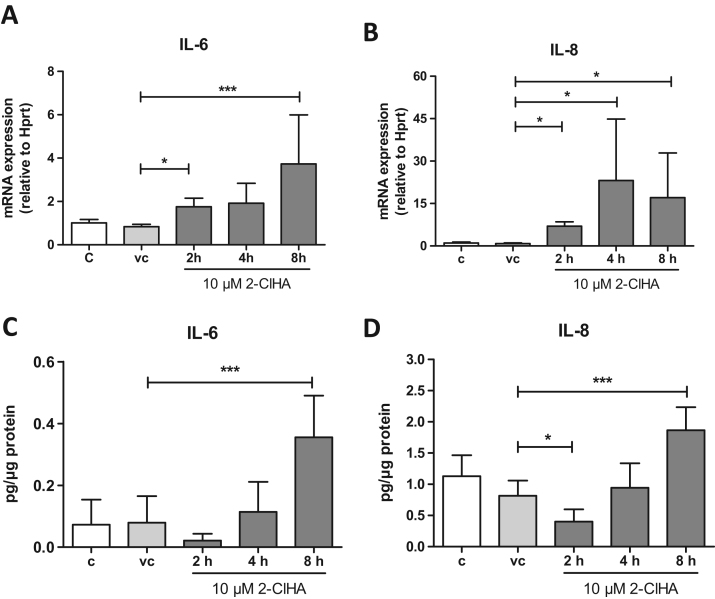
2-ClHA induces IL-6 and IL-8 gene and protein expression in hCMEC/D3 cells. (**A,B**) Serum-starved cells were incubated in the absence (c) or presence of DMSO (0.1%, vehicle control, vc) and 2-ClHA (10 μM) for the times indicated and gene expression of IL-6 (**A**) and IL-8 (**B**) was evaluated by qPCR analysis. Hprt was used as the housekeeping gene. Data shown are normalized to ‘vc’ and represent mean + SD from 3 independent experiments performed in triplicates. Expression profiles were calculated using the 2^-ddCt^ method. (**C,D**) Cells were treated as in (A) and IL-6 (**C**) and IL-8 (**D**) concentrations were quantitated in the cellular supernatants by ELISA. Results shown represent mean + SD from three independent experiments performed in triplicate. *, p < 0.05; ***, p < 0.001; one-way ANOVA with Bonferroni correction.

Inflammatory pathways in the CNS are linked to the ER stress response [41]. A previous study demonstrated the involvement of the pro-apoptotic transcription factor CHOP (an ATF4 target gene) in the regulation of pro-inflammatory cytokine secretion through intermediate NF-kB activation [42]. In line with this study, we found that treatment of hCMEC/D3 cells with 2-ClHA increased gene expression of IL-6 and IL-8 four- and forty-fold over baseline levels, respectively (Fig. 5**A,B**). Maximum upregulation of IL-6 was observed at 8 h while IL-8 was already fully induced at 4 h. Cytokine analysis in the cellular supernatants by ELISA confirmed the upregulation of IL-6 and IL-8 on protein level **(**Fig. 5**C,D)**.

**Fig. 6:**
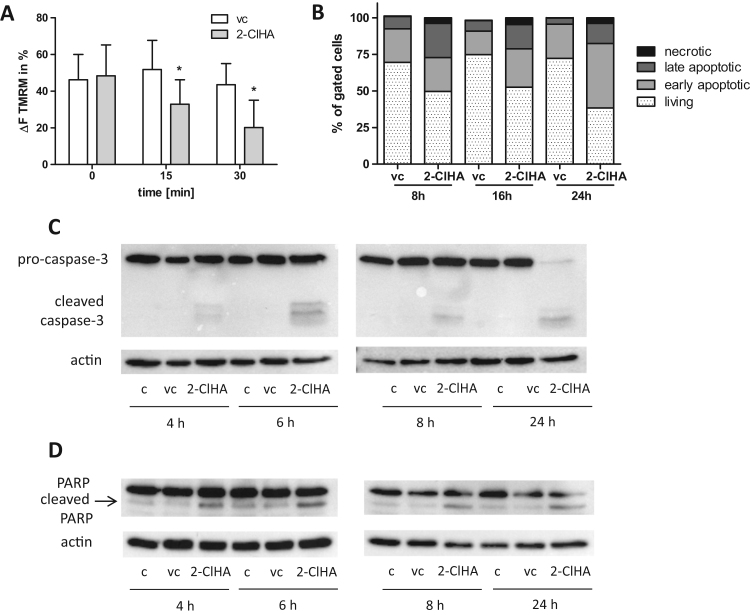
2-ClHA dissipates ΔΨ_m_ and induces apoptosis in hCMEC/D3 cells. Serum-starved cells were incubated in the presence of DMSO (0.1%, vehicle control, vc) and 2-ClHA (10 μM) for the indicated time points. **(A)** The bar graphs display quantification of TMRM fluorescence in vehicle (DMSO, 0.1%) and 2-ClHA-treated cells. Single cell measurements were background corrected and bleach corrected. The delta fluorescence (ΔF) intensity between basal and depleted membrane potential after FCCP treatment was measured. Results shown represent mean + SD. (**B**) Cells were trypsinized at the indicated time points, stained with Annexin V-FITC and propidium iodide (PI) and analyzed by flow cytometry. The bar graph summarizes the proportion of necrotic (A−/PI+), late apoptotic (A+/PI+), early apoptotic (A+/PI−), and living (A−/PI−) cells from two independent experiments performed in triplicates. Representative scattergrams are shown in Fig. S3. (**C,D**) At the indicated time points cells were lysed and (**C**) pro-caspase-3 and (**D**) PARP cleavage (arrow = 89 kDa product) was analyzed by Western blotting experiments. β-Actin was used as loading control. One representative blot out of three is shown. Densitometric and statistical evaluation is shown in Fig. S4.

Unmitigated ER stress can induce mitochondrial dysfunction and culminate in apoptosis to eliminate irreversibly damaged cells via apoptotic pathways [43]. These pathways are linked to a disturbed mitochondrial Ca^2+^ homeostasis, disruption of the mitochondrial membrane potential, and activation of pro-apoptotic caspases [44]. Analysis of the mitochondrial membrane potential (Ψ_m_) by cLSM using TMRM demonstrated that 2-ClHA caused a depolarization of Ψ_m_ indicating the induction of mitochondrial dysfunction (Fig. 6**A**).

To detect potential pro-apoptotic effects vehicle and 2-ClHA-treated cells were stained with Annexin V/PI and analyzed by flow cytometry. DMSO was without effect on early and late apoptosis, while 2-ClHA increased the percentage of early apoptotic cells from 24.7% to 47.6%; in parallel late apoptotic cells increased from 14% to 23% (scatter plots are shown in Fig. S3**;** statistical evaluation in Fig. 6**B**). In line, 2-ClHA activated caspase-3 (Fig. 6**C**), the convergence point of the extrinsic and intrinsic apoptotic machinery [45] and induced PARP cleavage (Fig. 6**D**; densitometric evaluations and statistical analyses are shown in Fig. S4).

**Fig. 7:**
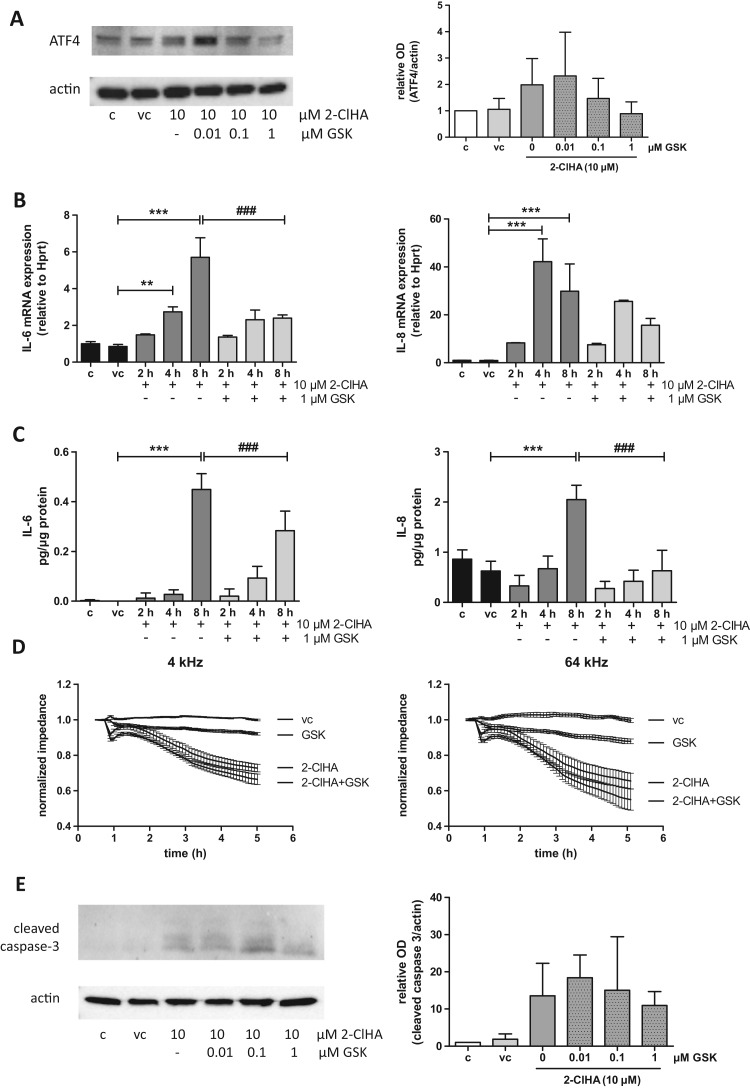
PERK inhibition attenuates the inflammatory response but not barrier dysfunction and caspase-3 activation of hCMEC/D3 cells. **(A)** Serum-starved cells were incubated in the absence (c) or presence of DMSO (0.1%, vehicle control, vc) and 2-ClHA in the absence or presence of different concentrations of GSK2606414 (GSK) for 4 h. Expression levels of ATF4 were examined by Western Blot analysis. One representative blot out of three is shown. Bar graph represents densitometric analyses of immunoreactive bands relative to β-actin (mean + SD; n = 3). **(B)** Serum starved cells were incubated in the absence (c) or presence of 2-ClHA plus GSK for the indicated time periods. DMSO (0.1%) served as vehicle control (vc). IL-6 (left) and IL-8 (right) mRNA levels were determined by qPCR normalized to Hprt. Values are expressed as mean + SD from three independent experiments performed in triplicates. Expression was calculated using the 2^-ddCt^ method. **(C)** Cells were treated as described in (B). The supernatants were collected and IL-6 (left) and IL-8 (right) concentrations were determined using ELISA. Results (normalized to protein content) represent mean + SD from three independent experiments performed in triplicates. (*, p < 0.01; one-way ANOVA with Bonferroni correction). **(D)** For ECIS measurements cells were plated on collagen-coated gold microelectrodes and cultured to confluence. After stabilization, DMSO (0.1%; vehicle control, vc) or 2-ClHA (10 μM) alone or in combination with 1 μM GSK was added to the cells. Impedance of cell monolayers was continuously monitored at 4 and 64 kHz. (**E**) Cells were treated as in (A). Caspase-3 cleavage (17 kDa) was evaluated by Western blot analysis (c = no addition; vc = DMSO). One representative Western blot out of three is shown. Densitometric and statistical evaluation is shown in Fig. S4.

The final set of experiments aimed to test whether pharmacological antagonism of PERK could suppress the inflammatory response and inhibit the induction of apoptosis. Therefore, hCMEC/D3 cells were pre-incubated with increasing GSK2606414 concentrations (0.01, 0.1, and 1 µM) and then received vehicle or 2-ClHA. Expression of ATF4 (which is downstream of PERK and induces pro-apoptotic CHOP) was followed by Western blot analysis. 2-ClHA-mediated ATF4 induction was attenuated by 1 µM GSK2606414 **(**Fig. 7**A**, densitometric evaluation in right bar graph). Real-time qPCR analyses demonstrated that GSK2606414 attenuated 2-ClHA-induced gene expression of IL-6 and IL-8 (Fig. 7**B**). GSK2606414 treatment also reduced IL-6 and IL-8 protein levels in the cellular supernatants (Fig. 7**C**). Finally, we addressed the question whether PERK inhibition can restore 2-ClHA-induced cell monolayer disintegration. Surprisingly, GSK2606414 did neither mitigate barrier dysfunction (Fig. 7**D**) nor pro-caspase-3 processing (Fig. 7**E**).

## 4 Discussion

MPO-mediated plasmalogen modification at the cerebrovascular interface results in the generation of reactive species including chlorinated fatty aldehydes and -acids that compromise cell [15], [32], [35], [46] and organ [22], [24] homeostasis. In the present study we have identified uptake of 2-ClHyA into the ER and mitochondria of brain endothelial cells. 2-ClHA interfered with palmitoylation, induced unresolved ER stress and mitochondrial dysfunction, culminating in apoptosis. Our data are in line with the fact that fatty acids are potent inducers of cell stress in the inflamed endothelium [47].

Although ample evidence indicates that MPO-derived oxidants play an important role in endothelial dysfunction [48] physiological relevance of 2-ClHA-induced BMVEC dysfunction is a major question arising from our in vitro study. The Ford group has shown that 2-chlorofatty acid levels in plasma of patients suffering from sepsis-associated acute respiratory distress syndrome are higher as compared to the control group (median concentrations 1.12 vs. 0.43 nM) [49]. At higher concentrations (up to 10 µM as used during the present study) 2-ClHA reduced pulmonary endothelial cell barrier function in vitro by approx. 25%. This observation was accompanied by enhanced cell adhesion molecule expression and increased neutrophil and platelet adherence [49]. However, it is important to note that the experiments described in [49] were performed in serum-containing medium (5%). Therefore the serum-free conditions used during the present study could generate a more ‘cytotoxic’ milieu since no 2-ClHA is bound by serum constituents via e.g. S-alkylation reactions.

Septic encephalopathy is a multifactorial syndrome, which is characterized as diffuse brain dysfunction in humans [50] and associated with neutrophil accumulation and BBB dysfunction in mice [51]. Our group could demonstrate that LPS-induced BBB dysfunction is accompanied by a decrease in plasmalogens while the corresponding MPO-derived oxidation product 2-ClHDA accumulated in brains of LPS-injected mice at a concentration up to 10 µM [14]. This is most probably a result of neutrophil accumulation and MPO release in the cerebrovasculature of mice in response to a systemic LPS injection [15]. Within this pathophysiological setting, BMVEC-adhering leukocytes could affect endothelial function via 2-ClHA production since both neutrophils and monocytes are able to produce high concentrations (in vitro up to 20 µM) of 2-ClHA [34], [35]. Finally, primary BMVEC and the hCMEC/D3 cell line used during the present study are able to convert exogenous 2-ClHDA to 2-ClHA [32], [52].

To determine intracellular localization of 2-ClHA we synthesized an alkyne-containing 2-ClHA analogue that allows covalent attachment of N_3_-containing reporter fluorophores by copper-catalyzed Huisgen 1.3-dipolar cycloaddition. During the synthesis of 2-ClHA or 2-ClHyA we utilized oxone, an oxidant offering several advantages (one step-one pot reaction, non-toxic, low cost reagent) over hazardous chromium(VI)-based systems like the highly toxic pyridinium dichromate [37], [53]. This synthetic route (starting from the corresponding aldehyde precursors 2-ClHDA or 2-ClHDyA, respectively) is straightforward, proceeds at moderate conditions (RT, 3 h) and the reaction yield and purity of the product is feasible (both >95%).

Subcellular localization of 2-ClHyA was determined by N_3_-TAMRA click-chemistry and subsequent visualization of compartment-specific markers by cLSM and SIM. These experiments revealed uptake of 2-ClHyA in the ER and mitochondria, raising the question of intracellular trafficking routes for this chlorinated fatty acid. Fatty acid transport proteins (FATP) are members of the Slc27 protein family with intrinsic acyl CoA synthase activity [54]. In the brain endothelium several membrane-associated FATPs were identified with highest expression reported for FATP-1, −4, and fatty acid translocase/CD36 [55]. In human carcinoma and canine kidney cells a major fraction of FATP4 was detected at the ER [56], [57]. In terms of mitochondrial 2-ClHA transport carnitine palmitoyltransferase 1 (CPT-1) and carnitine acylcarnitine translocase could facilitate mitochondrial import. Only recently the Ford group has shown that 2-ClHyA is localized to Weibel-Palade bodies and promotes the release of P-selectin, van Willebrand factor, and angiopoietin in human aortic endothelial cells [53].

ER and mitochondrial function play a critical role in neurodegenerative diseases. Although the crosstalk underlying ER stress-induced apoptosis is not completely understood, evidence suggests that cell survival vs. cell death decisions depend on mitochondrial Ca^2+^ handling [43]. Aberrant protein palmitoylation is implicated in the pathogenesis of neurodegenerative diseases, including Alzheimer's disease, Huntington's disease, or schizophrenia [58], and induces unresolved ER stress that culminates in cell death [59]. Pharmacological interference with S-palmitoylation is routinely performed with 2-BrHA, a chemical tool that inhibits palmitoyl acyl transferases but targets also other proteins by covalent alkylation [38], [60]. This is reminiscent of findings obtained during the present study: Using 2-ClHyA as activity-based probe, efficient protein labelling and only weak competition by HA was observed, findings that are compatible with non-specific covalent modification of target proteins (Fig. 3). This reaction most likely proceeds via chlorine abstraction at C2 and results in the formation of an irreversible and stable thioether adduct as described for 2-BrHyA [60]. Among 2-BrHA-modified proteins Davda and colleagues identified CPT-1 supporting mitochondrial accumulation of halogenated fatty acids [38] as also observed during the present study.

The ER depends on continuous supply of ATP to fulfill its biological functions [61]. Among these is the unfolded protein response that, under conditions of unresolved ER stress, represses an adaptive response and triggers apoptosis through activation of CHOP. Here, 2-ClHA upregulated the canonical PERK axis, namely eIF2α, ATF4, and CHOP, and decreased [ATP]_ER_. These findings were accompanied by Ψ_m_ dissipation. Altered Ca^2+^ homeostasis and dissipation of Ψ_m_ contributes to the opening of the mitochondrial permeability transition pore, which facilitates cytochrome c efflux-driven assembly of the apoptosome [45]. In line we observed increased pro-caspase-3 and PARP processing. This is reminiscent of what was reported for phorbolester-stimulated monocytes: In these cells 2-ClHA accumulates in response to activation and elicits apoptosis through generation of reactive oxygen species and ER stress [35].

In addition to ER stress 2-ClHA increased IL-6 and IL-8 on the mRNA and protein level. In vitro, IL-6 induces barrier dysfunction and increases IL-8 synthesis in human brain endothelial cells [62] as observed here in 2-ClHA-treated hCMEC/D3 cells. ER stress may contribute to sustained production of inflammatory mediators obstructing resolution of inflammation, a condition relevant to infectious, metabolic, and neurodegenerative diseases [63]. Human aortic endothelial cells upregulate synthesis of IL-6 and IL-8 in response to oxidized phospholipids in a PERK-dependent manner [64]. Of note, this class of oxidized lipids is not only detectable in atherosclerotic lesions [65] but also in brain of MS patients [66] and other settings of neurodegeneration [67]. Thus, blocking the inflammatory response through PERK inhibition could have pharmacological relevance in neurodegenerative diseases where ER stress is prevalent [43]. During the present study we used GSK2606414 (that also inhibits RIPK1; Ref. [68]) as PERK antagonist to reveal whether this compound would rescue 2-ClHA-induced brain endothelial dysfunction. We observed decreased ATF4 expression in response to GSK2606414 that also blunted the inflammatory response of BMVEC. However, GSK2606414 was without effect on pro-caspase-3 processing and associated barrier leakiness. This is reminiscent of what was reported for a panel of other ER stress inhibitors in Aβ1–40 treated rat brain endothelial cells [39].

In summary we identified 2-ClHA as an MPO-generated inflammatory trigger that induces ER stress and apoptosis in BMVEC. Our findings suggest that 2-chlorofatty acid generation during cerebrovascular inflammation holds potential to induce BBB dysfunction probably due to the local accumulation of these cytotoxic lipids in cellular organelles.
