# Supplementary material for: 2-Chlorohexadecanoic acid induces ER stress and mitochondrial dysfunction in brain microvascular endothelial cells
Source: Redox Biol. 2018 Jan 5;15:441–51. doi: 10.1016/j.redox.2018.01.003 (PMC5975063; doi:10.1016/j.redox.2018.01.003)
Supplement: Supplementary file 1 — Supplementary material [file mmc1.doc]

**SUPPLEMENTARY INFORMATION**

**2-Chlorohexadecanoic acid induces ER stress and mitochondrial dysfunction in cultured brain microvascular endothelial cells**

Eva Bernhart^1^, Nora Kogelnik^1^, Jürgen Prasch^1^, Benjamin Gottschalk^1^, Madeleine Goeritzer^1,2^, Maria Rosa Depaoli^1^**,** Helga Reicher^1^, Christoph Nusshold^3^, Ioanna Plastira^1^, Astrid Hammer^4^, Günter Fauler^5^, Roland Malli^1^, Wolfgang F. Graier^1,2^, Ernst Malle^1^, Wolfgang Sattler^1,2^

^1^ Gottfried Schatz Research Center for Signaling, Metabolism and Aging, Molecular Biology and Biochemistry, Medical University of Graz, Austria

^2^ BioTechMed Graz, Austria

^3^ Institute of Physiological Chemistry, Medical University of Graz, Austria

^4^ Gottfried Schatz Research Center for Signaling, Metabolism and Aging, Cell Biology, Histology and Embryology, Medical University of Graz, Austria

^5^ Clinical Institute of Medical and Chemical Laboratory Diagnostics, Medical University of Graz, Austria

**Materials**

Cell culture supplies were from Thermo Fisher Scientific (Waltham, MA US) and Sigma-Aldrich (St. Louis, MO, USA). **Hexadecan-1-oic acid (HA; palmitic acid),** dimethylsulfoxide (DMSO), pentafluorobenzyl (PFB) hydroxylamine, PFB bromide, *N,N*-Dimethylformamide (DMF), oxone, *N,N’*-diisopropylethylamine and 3-(4,5-dimethylthiazol-2-yl)-2,5-diphenyl tetrazolium bromide (MTT) were from Sigma-Aldrich. Hexadecanoic acid alkyne (15-HyA) was from Cayman (Ann Arbor, MI, USA). The protein kinase R-like ER kinase (PERK)-inhibitor GSK 2606414 was from Tocris Bioscience (Bristol, UK). Electrical cell-substrate impedance sensing (ECIS) electrode arrays (8W10E+) were from Ibidi (Martinsried, Germany). TransFast transfection reagent was from Promega (Madison, WI, USA). Click-iT© Cell and Protein Buffer Kits and antibody diluent were from Thermo Fisher Scientific (Vienna, Austria). 5-Tetramethylrhodamine azide (N_3_-TAMRA) was from Lumiprobe (Hannover, Germany). Ultra V blocking reagent was from Dako, Agilent (Santa Clara, CA, USA). Antibodies against calnexin, cytochrome c oxidase subunit 4 (COX IV), poly(ADP-ribose) polymerase (PARP), (phosphorylated)-eukaryotic translation initiation factor 2A ((p)EIF2α), 78 kDa glucose-regulated protein (BiP) and DNA damage-inducible transcript 3 protein (CHOP) were from Cell Signaling (New England Biolabs, Frankfurt, Germany). Polyclonal rabbit anti-caspase-3 antibody and anti activating transcription factor 4 (ATF4, CREB-2) antibodies were from Santa Cruz Biotechnology (Santa Cruz, CA, USA). Anti-β-actin antibody and horseradish peroxidase (HRP)-labeled secondary goat anti-rabbit IgG were from Sigma-Aldrich (St. Louis, MO, USA). Alexa 488-labelled goat anti-rabbit IgG was from Thermo Scientific. The FITC Annexin V Apoptosis Detection Kit 1 was from BD Biosciences (NJ, USA). Human IL-6 and IL-8 ELISA kits were from ImmunoTools (Friesoythe, Germany). Kits that were used for quantitative real-time PCR (qPCR) analysis were from QIAGEN (Hilden, Germany) or Applied Biosystems (Foster City, CA, USA). Primers were from Qiagen or Thermo Fisher Scientific (Invitrogen).

**Synthetic and analytical procedures**

Briefly, 1.5 mg 2-ClHDA (1 eq; or 2-chlorohexadec-15-yn-1-oic acid [2-ClHDyA] synthesized as described earlier [32]) was oxidized using 1.7 mg oxone (1 eq.) in 250 µl DMF (22 mM) at room temperature (RT) for 3 h (using a plateau shaker). The salts were dissolved in 250 µl HCl (1 M) and the product was extracted with hexane (2 times, 500 µl). The combined hexane phases were dried under a gentle stream of N_2_ and dried extracts were resuspended in 500 µl hexane followed by purification using silica gel column chromatography (hexane/diethyl ether, 90:10, v/v).

*Derivatization*

The products were converted to the corresponding PFB esters using 100 µl PFB-Br (0.35 %) in acetonitrile in the presence of 20 µl *N,N’*-diisopropylethylamine for 30 min at RT, dried under reduced pressure, resuspended in toluene (100 µl), transferred to autosampler vials, and stored at -20°C until negative ion chemical ionization gas chromatography-mass spectrometry (NICI-GC-MS) analysis.

*NICI-GC-MS analysis*

PFB-esters were separated on a Thermo Scientific Trace 1300 GC (helium was used as carrier gas, 2 ml/min) equipped with a SGE HT5 capillary column (25 m, 0.22 mm inner diameter, 0.1 μm phenyl polycarborane-siloxane film coating) and analyzed using an ISQ LT mass spectrometer (Thermo Scientific). Injector and ion source temperature were at 250°C. The oven temperature was maintained at 100°C for 5 min, increased during the first ramping step at a rate of 20°C/min to 175°C, and held for 1 min. In the second ramping step the temperature was raised at a rate of 15°C/min to 280°C and held for additional 2 min. All spectra were monitored in the NICI mode either in full scan mode or using selected ion monitoring using methane as reactant gas.

**Cell culture**

Human brain microvascular endothelial cells (hCMEC/D3; kindly provided by Dr. P.-O. Couraud; INSERM U1016 UMR 8104, Institut Cochin, Paris, France) were cultured in rat-collagen-coated 75 cm^2^ flasks in Earl’s salts-containing Medium 199 supplemented with 10% (v/v) fetal calf serum (FCS; GIBCO), 1% (v/v) streptomycin/penicillin, 1% (v/v) chemically defined lipid concentrate (Thermo), 1% (v/v) HEPES buffer, 1.4 μM hydrocortisone, 5 μg/ml ascorbic acid and 1 ng/ml bovine fibroblast growth factor at 37°C (5% CO_2_) until confluence. Prior to the corresponding experiments cells were serum-starved overnight in Earls salts-containing M199. Only passages below 38 were used for experiments. 2-ClHA was freshly diluted from a stock solution in DMSO. DMSO concentrations in the final medium never exceeded 0.1% (v/v). DMSO was used as vehicle control. To avoid trapping of chlorinated fatty acids by serum proteins all experiments were conducted with serum-starved cells.

#### 3-(4,5-Dimethyl-2-thiazolyl)-2,5-diphenyl-2H-tetrazolium bromide (MTT) test

The metabolic activity of hCMEC/D3 cells treated with 2-ClHA and its alkyne derivative 2-ClHyA was assessed using the MTT assay (Sigma Aldrich, Vienna, Austria). Cells seeded in collagen-coated 48-well plates were grown to confluence, serum-starved overnight and treated with 10 µM of 2-ClHA or 2-ClHyA for the indicated time periods. Then, cells were incubated with MTT (1.2 mM in serum-free medium) for 1 h, washed with PBS and lysed with isopropanol/1 M HCl (25:1; v/v) on a rotary shaker at 1200 rpm for 15 min. Absorbance was measured at 570 nm on a Victor 1420 multilabel counter (Wallac) and corrected for background absorption (650 nm).

**Electrical cell-substrate impedance sensing** **(ECIS)**

To assess the effects of HA, 2-ClHA, 2-ClHyA, and the PERK inhibitor GSK2606414 on barrier function, impedance monitoring was performed with an ECIS Z system (Applied Biophysics, Troy, NY, USA). Cells were seeded on collagen-coated gold electrodes of 8W10E+ arrays, cultured until confluence and serum-starved overnight prior to the experiment. For inhibition studies, cells were pre-treated with GSK2606414 (1 µM) for 3 h and then co-incubated with 10 µM of 2-ClHA. Impedance was recorded in real time at 1 min intervals at 4 kHz (barrier function) and 64 kHz (cell viability and monolayer integrity).

##### **Metabolic labelling, click-chemistry and confocal microscopy**

For immunofluorescence microscopy, hCMEC/D3 cells plated onto collagen-coated coverslips were grown to confluence, serum starved for 3 h and incubated in the presence of 25 µM 2-ClHyA for 30 min. After washing with PBS, cells were fixed with 3.7 % paraformaldehyde in PBS for 15 min and permeabilized with 0.25% (v/v) Triton X-100 in PBS for 15 min at RT. Subsequently, cells were washed with 1% (w/v) BSA in PBS and 2-ClHyA-containing proteins were labelled with N_3_-TAMRA using the Click-iT® Cell Reaction Buffer Kit according to the manufacturer`s recommendations (Life Technologies). Following click reaction, cells were washed twice with PBS and nonspecific absorption was blocked with Ultra V blocking reagent (Dako) for 10 min. Then, cells were incubated at 4°C overnight with antibodies against COX IV or Calnexin (1:100 in antibody diluent). Alexa488-labeled goat anti-rabbit IgG (1:300 in antibody diluent) was used as secondary antibody. Confocal laser scanning microscopy (cLSM) was performed using a Leitz/Leica TCSSP2 microscope (Leica Lasertechnik GmbH, Heidelberg, Germany) or a Zeiss Observer Z.1 inverted microscope equipped with a Yokogawa CSU-X1 Nipkow spinning disk system, a piezoelectric z-axis motorized stage (CRWG3-200; Nippon Thompson Co., Ltd., Tokyo, Japan), and a CoolSNAP HQ2 CCD Camera (Photometrics). Alexa 488 and TAMRA fluorescence signal was acquired sequentially.

**High-Resolution Structured Illumination Microscopy (SIM)**

For ER-staining the ER-localized ATP sensor ERAT4.01 was used (transfection details are given in ‘ER ATP measurements’). Click-chemistry and COX IV staining was performed as described above. The SIM-setup used is composed of a 405 nm, 488 nm, 515 nm, 532 nm and a 561 nm excitation laser introduced at the back focal plane inside the SIM-box with a multimodal optical fiber. For super-resolution, a CFI SR Apochromat TIRF 100x-oil (NA 1.49) objective was mounted on a Nikon-Structured Illumination Microscopy (N-SIM®) System with standard wide field and SIM filter sets and equipped with two Andor iXon3® EMCCD camera mounted to a Two Camera Imaging Adapter (Nikon Austria, Vienna, Austria). For calibration and reconstruction of SIM images the Nikon software Nis-Elements was used.

**Mitochondrial Membrane Potential (Ψ_m_)**

Cells were incubated for 20 min in 120 nM TMRM (Tetramethylrhodamine methyl ester perchlorate, Invitrogen^TM^) in loading-buffer containing 2 mM CaCl_2_, 135 mM NaCl, 5 mM KCl, 1 mM MgCl_2_, 1 mM HEPES, 2.6 mM NaHCO_3_, 0.44 mM KH_2_PO_4_, 0.34 mM Na_2_HPO_4_, 10 mM D-glucose (Roth), 0.1% vitamins, 0.2% essential amino acids and 1% penicillin/streptomycin (GibcoTM) at pH 7.4. Cells were washed once with loading-buffer and incubated for 0, 15 or 30 min with 2-ClHA or DMSO (as vehicle control). During cLSM the probes were excited via a polychrome V (Till Photonics) and emission was visualized using a 40x objective (alpha Plan Fluar 40, Zeiss, Goettingen, Germany), and a charge-coupled device camera (AVT Stringray F145B, Allied Vision Technologies, Stadtroda, Germany). TMRM was excited at 550 nm, and emissions were captured at 600 nm (59004; Chroma, Bellows Falls, VT, USA). Cells were treated with 5 µM FCCP (Carbonyl cyanide *p*-trifluoromethoxyphenylhydrazone, Sigma Aldrich) after 2 min. Single cell measurements were background corrected and bleach corrected. The delta fluorescence intensity between basal and depleted membrane potential 6 min after FCCP treatment was measured.

##### **Metabolic labelling, click-chemistry and 1D SDS-PAGE analysis**

Confluent hCMEC/D3 cells in collagen-coated 6-well dishes were serum-starved overnight and treated with the indicated concentrations of HA, hexadec-15-yn-1-oic acid (HyA), 2-ClHA, or 2-ClHyA for 4 h. After washing with ice-cold PBS, cells were scraped off in 25 µl lysis buffer (50 mM Tris/HCl, 1% SDS, pH 8.0) containing protease and phosphatase inhibitors and 250 U/ml Benzonase (Merck, Darmstadt, Germany). Lysates from two 6-well culture dishes were pooled, sonicated, vortexed (5 min) and centrifuged (18,000x*g*, 5 min, 4°C). Equal amounts of alkyne-labelled protein (150–200 µg in a maximum volume of 50 µl lysis buffer) were subjected to click reaction using the Click-iT® Protein Reaction Buffer Kit (Life Technologies) and subsequent SDS-PAGE according to the manufacturer`s instructions. N_3_-TAMRA was used as fluorophore for protein detection via fluorescence imaging using a Typhoon 9400 scanner (Amersham Biosciences; excitation 532 nm, emission 580 nm). Coomassie Brilliant Blue staining was performed to verify equal loading. Fluorescence and Coomassie intensities were analyzed using ImageQuant and Image Lab 4.0.1 software, respectively.

**Western blot analysis**

For immunoblotting, hCMEC/D3 cells were seeded onto 6-well plates, grown until confluence, and incubated in serum-free M199 medium overnight before starting the experiment. Cells were stimulated with 2-ClHA at the indicated concentrations in the absence or presence of GSK2606414 (concentrations as indicated; 3 h pre-incubation) for various time periods. After removing the supernatant, cells were washed two times with ice-cold PBS and lysed using RIPA buffer (50 mM Tris-HCl pH 7.4, 1% NP-40, 150 mM NaCl, 1 mM Na_3_VO_4_, 1 mM NaF, 1 mM EDTA) including protein inhibitors (aprotinin, leupeptin, pepstatin [1 μg/ml each] and 10 μM PMSF). Cells were lysed by mechanically scrapping and centrifuged at 13,000 rpm for 10 min. Protein estimation of whole cell lysates was determined using a BCA kit (Thermo Scientific). Forty μg of total cellular protein was separated by SDS-PAGE and transferred to PVDF membranes. Membranes were blocked with 5% low fat milk in TBS containing Tween20 and probed with the following antibodies: anti-ATF4 (1:1000), anti-p-eIF2α (1:500), anti-BiP (1:1000), anti-CHOP (1:1000), anti-caspase-3 (1:1000) or anti-PARP (1:1000). Immunoreactive bands were visualized using HRP-conjugated goat anti-rabbit IgG (1:5000) and subsequent chemiluminescence HRP substrate development (Immobilon, Millipore). For normalization, membranes were stripped and re-probed with primary antibodies against non-phosphorylated eIF2α (1:1000) or β-actin (1:5000). Immunoreactive bands were visualized using the chemiluminescence detection system ChemiDoc (Bio-Rad, Berkeley, CA, USA).

**ER ATP measurements using a genetically encoded sensor**

hCMEC/D3 cells were plated onto collagen-coated 30 mm glass coverslips and cultured until 60-80% confluence. Then, cells were transfected with 1.5 µg of the FRET-based ATP sensor ERAT4.01 [36] and 3 µl of TransFast transfection reagent (Promega) in 1 ml of serum- and antibiotic-free medium for 4 h, followed by 36 h cultivation in full medium. Prior to the measurement cells were kept in loading buffer containing 135 mM NaCl, 5 mM KCl, 2 mM CaCl_2_, 1 mM MgCl_2_, 10 mM Hepes, 2.6 mM NaHCO_3_, 440 mM KH_2_PO_4_, 340 mM Na_2_HPO_4_, 10 mM D-glucose, 0.1% vitamins, 0.2% essential amino acids, and 1% penicillin–streptomycin, pH 7.4. Following incubation with 25 µM of 2-ClHA or HA for 30 min in loading buffer, fluorescent recordings were performed using an advanced wide-field fluorescent microscope (Till Photonics, Graefling, Germany) equipped with a motorized sample stage, a polychrome V (Till Photonics), a 40x objective (alpha Plan Fluar 40x, Zeiss, Göttingen, Germany), and a charge-coupled device camera (AVT Stingray F145B, Allied Vision Technologies, Stadtroda, Germany). The FRET-based ER ATP sensor ERAT4.01 excited at 430 nm and emission was collected using the dichrotome dual emission filter set (dichroic 535dcxr, CFP emitter 482/18 nm and YFP emitter 535/3 nm). Data acquisition and stage control was carried out by the Live Acquisition 2.0.0.12 software (Till Photonics). Results of FRET measurements are shown as ratio (cell fluorescence 535 nm - background 535 nm)/(cell fluorescence 480 nm - background).

**Annexin V/propidium iodide (PI) staining**

hCMEC/D3 cells were seeded onto rat collagen-coated 6-well plates and serum-starved overnight prior to the experiments. Then, cells were treated with 10 µM 2-ClHA for the indicated times. Next, cells were collected and stained using the FITC Annexin V Apoptosis Detection Kit (BD Biosciences, NJ, US) according to the manufacturer`s protocol.

Flow cytometry analysis was performed using a Guava EasyCyte 8 (Millipore, Billerica, MA, USA) and analysed using InCyte 3.1 (Millipore). For fluorescence compensation and gating, unstained and single-stained cells were treated with either staurosporine (3 μM; 6 h) or H_2_O_2_ (3 mM; 4 h) in order to detect apoptotic and necrotic cells, respectively.

**qPCR analysis**

For mRNA quantitation, confluent serum-starved hCMEC/D3 cells were stimulated with 10 μM 2-ClHA in the absence or presence of GSK2606414 (1 µM; 3 h pre-incubation) followed by total RNA isolation using the RNeasy Mini Kit (Qiagen, Hilden, Germany). Total RNA was quantitated via NanoDrop (Thermo Fisher Scientific, Waltham, MA, USA) and reverse-transcribed using the high-capacity cDNA reverse transcription kit (Applied Biosystems, Foster City, CA, USA). Quantitative real-time PCR was carried out on an Applied Biosystems 7900HT Fast Real-Time PCR system using QuantifastTM SYBR Green PCR. Relative gene expression was normalized to hypoxanthine-guanine phosphoryltransferase (Hprt). Expression profiles and associated statistical parameters were analysed by the 2-ddCt method. Primer sequence IL-6 fwd: GGTACATCCTCGACGGCATCT; IL-6 rev: GTGCCTCTTTGCTGCTTTCAC; IL-8 fwd: AAGAGAGCTCTGTCTGGACC; lL-8 rev: GATATTCTCTTGGCCCTTGG

**ELISA**

Concentrations of IL-6 and IL-8 in the cellular supernatants were determined using human ELISA kits (ImmunoTools, Friesoythe, Germany). hCMEC/D3 cells were seeded onto rat collagen-coated 6-well plates and grown to confluence. Cells were serum-starved over night and stimulated with 10 μM 2-ClHA in the absence or presence of 1 μM GSK2606414 for indicated time periods including a 3 h pre-treatment with GSK2606414. Cellular supernatants were collected, centrifuged, snap frozen in liquid nitrogen, and stored at -80°C until analysis. ELISAs were performed according to the manufacturer’s protocol. Protein concentration of cell lysates was determined using the BCA assay and cytokine concentrations were normalized to the corresponding protein values. All samples were analyzed in triplicate.

**PERK inhibition studies**

To investigate the involvement of PERK on ATF4 expression and cytokine synthesis/secretion in response to 2-ClHA treatment, PERK was pharmacologically antagonized with GSK2606414 (10 - 1000 nM). hCMEC/D3 cells were serum-starved, pre-incubated with GSK2606414 for 3 h, and treated with 10 µM 2-ClHA in the presence of GSK2606414 for the indicated time periods.

#### Statistical analyses

Data are presented as means ± SD. To test differences in groups, statistical significance was determined by one-way ANOVA with Bonferroni correction (using the GraphPad 5.0 Prism package) as indicated. All values of p≤0.05 were considered significant. *, p<0.05, **, p<0.01, ***, p<0.001; * compared to vehicle; # compared to the same concentration of 2-ClHA.

**References:**

[32] Nusshold, C.; Ullen, A.; Kogelnik, N.; Bernhart, E.; Reicher, H.; Plastira, I.; Glasnov, T.; Zangger, K.; Rechberger, G.; Kollroser, M.; Fauler, G.; Wolinski, H.; Weksler, B. B.; Romero, I. A.; Kohlwein, S. D.; Couraud, P. O.; Malle, E.; Sattler, W. Assessment of electrophile damage in a human brain endothelial cell line utilizing a clickable alkyne analog of 2-chlorohexadecanal. Free Radic Biol Med 90:59-74; 2016.

[36] Vishnu, N.; Jadoon Khan, M.; Karsten, F.; Groschner, L. N.; Waldeck-Weiermair, M.; Rost, R.; Hallstrom, S.; Imamura, H.; Graier, W. F.; Malli, R. ATP increases within the lumen of the endoplasmic reticulum upon intracellular Ca2+ release. Mol Biol Cell 25:368-379; 2014.

**Fig. S1:**

**(A)** MTT reduction by hCMEC/D3 cells incubated in the absence (control; c) or presence of DMSO (0.1%, vehicle control, vc), 2-ClHA or 2-ClHyA in DMSO (both 10 µM) for the indicated times. MTT reduction by 2-ClHA and 2-ClHyA treated cells is expressed as % of vc. *, p<0.05; ***, p<0.001; one-way ANOVA with Bonferroni correction compared to vehicle.

**(B)** For ECIS measurements, cells were plated on collagen-coated gold microelectrodes and cultured to confluence. After baseline stabilization, 2-ClHA, 2-ClHyA, HA, or DMSO (vc; 0.1 %) were added to the cells. Impedance of cell monolayers was monitored at 4 and 64 kHz for the time indicated in the absence (c; control) or presence of the indicated compounds.

**Fig. S2:**

Densitometric evaluation of immunoreactive p-eIF2α, ATF4, CHOP, and BiP. hCMEC/D3 cells were incubated in the absence (control; c) or presence of DMSO (0.1%, vehicle control, vc) or 2-ClHA in DMSO (10 µM; ‘2-ClHA’) for the indicated times. Values are expressed as mean+SD (n=3) and normalized to actin as loading control. *, p<0.05; **, p<0.01; one-way ANOVA with Bonferroni correction. OD = optical density.

**Fig. S3:**

hCMEC/D3 cells were incubated with DMSO (0.1 % v/v; upper panel) or 2-ClHA (10 μM, lower panel) for the indicated time periods. Cells were trypsinized, stained with Annexin V-FITC and PI and analyzed by flow cytometry. Representative scattergrams are shown. To set up fluorescent compensation and gating, unstained and single-stained positive controls (3 μM staurosporine, 6 h for apoptotic cells or 3 mM H_2_O_2_, 4 h for necrotic cells) were used.

**Fig. S4:**

Densitometric evaluation of immunoreactive cleaved caspase-3 and PARP. hCMEC/D3 cells were incubated in the absence (control; c) or presence (0.1%, vehicle control, vc) of DMSO or 2-ClHA in DMSO (10 µM; ‘2-ClHA’) for the indicated times. Values are expressed as mean+SD (n=3) and normalized to actin as loading control. *, p<0.05; ***, p<0.001; one-way ANOVA with Bonferroni correction. OD = optical density.
